# P-1064. Safety, Tolerability, and Pharmacokinetics of Rifaquizone for Injection in Healthy Participants in China: A Single-center, Double-blind, Randomized, Placebo-controlled, Phase 1 Bridge Study

**DOI:** 10.1093/ofid/ofae631.1253

**Published:** 2025-01-29

**Authors:** Xiaojie Wu, Jing Zhang, Qiong Wei, Nanyang Li, Sung Min Park, Navaldeep Singh, Jing Chen, Changlin Ai, Guozhu Geng, Quan Zhang, Zhenkun Ma

**Affiliations:** Clinical Pharmacology Research Center, Huanshan Hospital, Shanghai, Shanghai, China (People's Republic); Huashan Hospital, Fudan University, Shanghai, Shanghai, China; Clinical Pharmacology Research Center, Huanshan Hospital, Shanghai, Shanghai, China (People's Republic); Huashan Hospital, Fudan University, Shanghai, Shanghai, China; Clinical Pharmacology Modeling and Simulation, Allucent, Cary, North Carolina; Clinical Pharmacology Modeling and Simulation, Allucent, Cary, North Carolina; TenNor Therapeutics (Suzhou) Ltd, Suzhou, Jiangsu, China (People's Republic); TenNor Therapeutics (Suzhou) Ltd, Suzhou, Jiangsu, China; TenNor Therapeutics (Suzhou) Ltd, Suzhou, Jiangsu, China; TenNor Therapeutics (Suzhou) Ltd, Suzhou, Jiangsu, China; TenNor Therapeutics, Suzhou Industrial Park, Jiangsu, China (People's Republic)

## Abstract

**Background:**

Rifaquizinone (RFQ, TNP-2092) is a novel multitargeting antibacterial agent under clinical development for the treatment of acute bacterial skin and skin structure infections (ABSSSIs) and prosthetic joint infections (PJIs). This study assessed the safety, tolerance, and pharmacokinetics (PK) of RFQ for injection in healthy Chinese participants and compared PK profile between Chinese and the US population.

**Figure:**
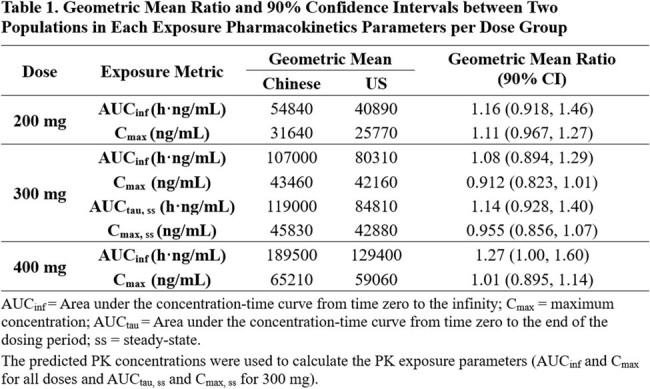

**Methods:**

A randomized study was conducted in a single center in China (NCT06394518). Thirty-two eligible participants were enrolled into 3 study cohorts, receiving 200, 300, or 400 mg of RFQ intravenously. In the 200 mg and the 400 mg cohorts, 8 participants in each cohort were randomized in a 3:1 ratio to receive a single intravenous (IV) infusion of RFQ or placebo. In the 300 mg cohort, 16 participants were randomized in a 3:1 ratio to receive a single dose of RFQ or placebo on Day 1 and then to receive multiple doses of RFQ or placebo q12h for 7 days starting on Day 4. The safety and tolerability were assessed, and the PK parameters were calculated. A population PK (popPK) model was developed to evaluate the differences of the Chinese and the US populations.

**Results:**

Thirteen participants experienced adverse events (AEs), all of which were mild or moderate in severity. Following a single dose of RFQ, the mean maximum observed concentration (C_max_) increased linearly, while the area under concentration-time curve (AUC) increased slightly higher than the dose proportion. Following 7 days of multiple doses of RFQ q12h, the steady state was reached after 4 doses with slight drug accumulation observed. Based on popPK analysis, AUC and Cmax were calculated from the predicted PK concentrations, and a 13.9% reduction in clearance (CL) in Chinese population was observed compared to US population. However, the 90% confidence interval (CI) of the geometric mean ratios (GMRs) of AUC and C_max_ essentially included 1.0, indicating similarities in drug exposure between the two populations.

**Conclusion:**

RFQ for injection was well tolerated in healthy Chinese participants. The C_max_ was proportional to dose, while the AUC increased slightly higher than the dose proportion. The exposure between the Chinese and the US populations was not significantly different.

**Disclosures:**

**Xiaojie Wu, PhD**, TenNor Therapeutics (Suzhou) Ltd: Investigator **Jing Zhang, Doctor of Medicine**, TenNor Therapeutics (Suzhou) Ltd: Investigator **Qiong Wei, Bachelor**, TenNor Therapeutics (Suzhou) Ltd: Investigator **Nanyang Li, Master of Medicine**, TenNor Therapeutics (Suzhou) Ltd: Investigator **Sung Min Park, PhD**, TenNor Therapeutics (Suzhou) Ltd: Pharmacological statistician **Navaldeep Singh, PharmaD**, TenNor Therapeutics (Suzhou) Ltd: Pharmacological statistician **Jing Chen, Master of Science**, TenNor Therapeutics (Suzhou) Ltd: Employee **Changlin Ai, Master of Medicine**, TenNor Therapeutics (Suzhou) Ltd: Employee **Guozhu Geng, MD**, TenNor Therapeutics (Suzhou) Ltd: Employee **Quan Zhang, Bachelor**, TenNor Therapeutics (Suzhou) Ltd: Employee **Zhenkun Ma, PhD**, TenNor Therapeutics (Suzhou) Ltd: Employee

